# Comparative scaffolding and gap filling of ancient bacterial genomes applied to two ancient *Yersinia pestis* genomes

**DOI:** 10.1099/mgen.0.000123

**Published:** 2017-07-08

**Authors:** Nina Luhmann, Daniel Doerr, Cedric Chauve

**Affiliations:** ^1^​ International Research Training Group "Computational Methods for the Analysis of the Diversity and Dynamics of Genomes", Bielefeld University, Bielefeld, Germany; ^2^​ Genome Informatics, Faculty of Technology and Center for Biotechnology, Bielefeld University, Bielefeld, Germany; ^3^​ School of Computer and Communication Sciences, EPFL, 1015 Lausanne, Switzerland; ^4^​ Department of Mathematics, Simon Fraser University, Burnaby, BC, Canada

**Keywords:** ancestral reconstruction, comparative genomics, assembly, *Yersinia pestis*

## Abstract

*Yersinia pestis* is the causative agent of the bubonic plague, a disease responsible for several dramatic historical pandemics. Progress in ancient DNA (aDNA) sequencing rendered possible the sequencing of whole genomes of important human pathogens, including the ancient *Y. pestis* strains responsible for outbreaks of the bubonic plague in London in the 14th century and in Marseille in the 18th century, among others. However, aDNA sequencing data are still characterized by short reads and non-uniform coverage, so assembling ancient pathogen genomes remains challenging and often prevents a detailed study of genome rearrangements. It has recently been shown that comparative scaffolding approaches can improve the assembly of ancient *Y. pestis* genomes at a chromosome level. In the present work, we address the last step of genome assembly, the gap-filling stage. We describe an optimization-based method AGapEs (ancestral gap estimation) to fill in inter-contig gaps using a combination of a template obtained from related extant genomes and aDNA reads. We show how this approach can be used to refine comparative scaffolding by selecting contig adjacencies supported by a mix of unassembled aDNA reads and comparative signal. We applied our method to two *Y. pestis* data sets from the London and Marseilles outbreaks, for which we obtained highly improved genome assemblies for both genomes, comprised of, respectively, five and six scaffolds with 95 % of the assemblies supported by ancient reads. We analysed the genome evolution between both ancient genomes in terms of genome rearrangements, and observed a high level of synteny conservation between these strains.

## Abbreviations

aDNA, ancient DNA; CAR, contiguous ancestral region; IS, Insertion sequence.

## Data Summary

1. The implementation of the AGapEs method is available at GitHub (http://github.com/nluhmann/AGapEs).

2. The data underlying the following results can be downloaded from http://paleogenomics.irmacs.sfu.ca/DOWNLOADS/AGAPES_data_results.zip.

3. Twelve supplementary tables and twenty supplementary figures are available with the online Supplementary Material.

## Impact Statement

In this work, we present a method to improve the scaffolding and gap filling of fragmented ancient DNA (aDNA) assemblies using a combination of a template obtained from related extant genomes and aDNA reads. We obtained highly improved genome assemblies for two data sets of *Yersinia pestis* from victims of the London Plague in the 14th century and of the Marseille Plague in the 18th century. Our comparative analysis of the improved assemblies led to the surprising observation of high genome structure conservation, despite a time difference of almost 400 years between the ancient strains and a high rate of genome rearrangements in the *Y. pestis* family.

## Introduction

*Yersinia pestis* is the pathogen responsible for the bubonic plague, a disease that marked human history through several dramatic pandemics, including the Justinian Plague and the Black Death. It diverged a few thousand years ago from a relatively non-virulent pathogen, *Yersinia pseudotuberculosis*. The precise timing of the divergence between these two pathogens is still controversial [1], but it is widely accepted that the emergence of *Y. pestis* as a virulent human pathogen was characterized, among other elements, by the acquisition of numerous repeat sequences, especially insertion sequences (ISs) that triggered an extensive chromosomal rearrangement activity [2, 3]. Also worth noting, loss-of-function mutations that can be caused by chromosomal rearrangements have been identified as evolutionary adaptations for flea-borne transmission from *Y. pseudotuberculosis* in the ecological context [4]. This makes the family *Yersinia* appear to be an interesting model for the study of genome rearrangements during pathogen evolution.

Traditionally, the study of genome rearrangements relies on a comparative approach using the genomes of related extant organisms. Under appropriate models of evolution, this comparison provides indirect insight into genomic features of ancient species and their evolution toward extant species, see Darling *et al*. [2] for example for the specific case of genome rearrangements in *Yersinia*. However, this approach requires well-assembled extant genomes, as otherwise it is difficult to distinguish breakpoints due to assembly fragmentation from evolutionary breakpoints. For example, Auerbach *et al*. [5] discussed several chromosomal rearrangements between two closely related *Y. pestis* strains, but could not determine the evolutionary history of these modifications as related strains were only partially assembled and highly rearranged. Besides challenges for the analysis of genome rearrangements, fragmented assemblies of bacterial genomes impede subsequent analysis like genome annotation, the identification of gene duplication, gene loss and lateral gene transfer, or the characterization of gene families, as well as the analysis of intergenic and especially repeat-rich genomic regions, which are usually not assembled [6–9]. Finally, while synteny breakpoints often coincide with gaps in a conservative assembly, unfinished assemblies also pose the jeopardy of uncorrected mis-assemblies influencing the reconstruction of genome rearrangement events [10, 11].

In contrast to the approach based on comparing extant genomes, sequenced ancient DNA (aDNA) extracted from conserved remains can give direct access to the sequence of ancient genomes and, thus, theoretically, allows us to study the evolution from ancestors to descendants directly. Following advances in aDNA high-throughput sequencing technologies and protocols [12–17], the genomes of several ancient human, animal and plant pathogens have recently been sequenced at the level of complete or almost complete chromosomes, including the agents of potato blight [18, 19], brucellosis [20], tuberculosis [21] and leprosy [22], *Helicobacter pylori* [23], and the agents of cholera [24] and the bubonic plague [25–27], leading to important historical and evolutionary discoveries. However, unlike extant DNA high-throughput sequencing, which is experiencing a breakthrough transition towards long-reads, aDNA sequencing methods generate extremely short reads with low and non-uniform coverage [14]. As a result, aside of rare exceptions [22], the assembly of aDNA reads generates numerous short contigs. For example, a reference-based assembly of the Black Death pandemic agent resulted in several thousand contigs [26], two thousand of them of length 500 bp and above. While short aDNA reads can be mapped onto one or several extant reference genomes to detect important evolutionary signals such as Single Nucleotide Polymorphisms (SNPs) and small indels [28, 29], fragmented assemblies make it challenging to exploit aDNA sequencing data similar to fragmented assemblies of extant strains to analyse the evolution of pathogen genome organization.

Without long-read sequencing data, comparative scaffolding based on the comparison of the contigs of a genome of interest with related assembled genomes has proven to be a useful approach to improve the assembly of fragmented genomes, especially bacterial genomes [30–33]. Among such methods, FPSAC [31] was introduced to improve ancient genome assemblies within a phylogenetic context. It was applied to aDNA contigs from the *Y. pestis* strain responsible for the medieval London bubonic plague outbreak – that was shown to be ancestral to several extant *Y. pestis* strains [26] – and resulted in an improvement of the initial contig assembly from thousands of contigs to a chromosome-scale scaffolding. Moreover, taking advantage of the high sequence conservation in *Y. pestis* genomes, the inter-contigs gaps of the ancient *Y. pestis* strain were filled with putative sequences reconstructed from multiple sequence alignments of conserved extant gaps. This gap-filling step shed an interesting light on genomic features hidden within the assembly gaps, in particular Insertion Sequences (ISs) and their correlation with rearrangement breakpoint reuse, but also allowed the potential reconstruction of regions that were not recovered or were absent from the aDNA material. However, the scaffolding of adjacencies and gap sequences obtained in this previous work [31], which accounted for roughly 20 % of the genome size, were inferred through computational methods within a parsimony framework, which can be sensitive to convergent evolution that cannot be ruled out for genomes with a high rate of genome rearrangements, such as *Y. pestis* [2].

In the present work, we address this issue by using the large set of aDNA reads that are unassembled after the contig assembly stage, to confirm the scaffolding of contigs as well as sequences for inter-contigs gaps. We introduce the method AGapEs (Ancestral Gap Estimation), which attempts to fill the inter-contig gap between two adjacent ancient contigs by selecting a set of overlapping aDNA reads that minimizes the edit distance to a template gap sequence obtained from the extant genome sequences that support the adjacency. We directly included annotations of potential ISs in the extant genomes in the analysis to use the aDNA reads when the presence of an IS in the ancient genome is doubtful due to a mixed signal of presence/absence in the supporting extant genomes.

We have applied this strategy to two data sets of aDNA reads for ancestors of the human pathogen *Y. pestis* [3, 34]. The first aDNA data was obtained from a London victim of the Black Death pandemic in the 14th century [26], and the second consisted of five samples from victims of the Great Plague of Marseille around 400 years later [27]. For both data sets, we obtained an assembly with reduced fragmentation and were able to fill a large number of inter-contig gaps with aDNA reads. We identified several genome rearrangements between the ancient strains and extant *Y. pestis* genomes; however, observed only a single small inversion between both ancient strains, suggesting that the genome organization of the agent of the second major plague pandemic was highly conserved.

## Methods

Herein, we first describe AGapEs, the novel gap-filling method we introduce, followed by a description of the data and pre-processing steps for our analysis of two ancient *Y. pestis* strains.

### AGapEs: assembly of ancestral gap sequences from aDNA reads

The main methodological contribution we introduce is a template-based method to assess the validity of a potential ancestral adjacency through gap-filling. The general principle is to associate to every ancestral gap a template sequence obtained from the supporting extant gap sequences. We can then map aDNA reads onto this template and assemble the mapped aDNA reads into a sequence that minimizes the edit distance to the template sequence. The rationale for this template-based approach is that, due to the low coverage of the aDNA reads and their short length, existing gap-filling methods might fail to fill a large number of ancestral gaps.

#### Input

AGapEs takes as input a set of aDNA reads, together with a set of aDNA contigs, a set of assembled extant reference genomes and a phylogenetic tree relating all considered species.

#### Computing orthologous marker families

A family of orthologous *markers* is a set of genomic sequences, one from each extant genome and one from the ancient genome of interest. They are assumed to be highly similar, suggesting they evolved from a single ancestral genomic locus. So, each marker family contains one marker per extant genome (called extant markers from now on) and one ancient marker. Each extant marker is naturally associated with the genomic coordinates and orientation of its locus along the corresponding extant genome. This orientation of extant markers allows us to define two extremities for each sequence, namely its tail (5' extremity) and its head (3' extremity). Assuming an unassembled ancient genome, ancient markers are not associated to genome coordinates. Given the aDNA contigs and extant reference genomes, marker families are computed using the FPSAC algorithm [31]: the aDNA contigs are aligned onto each of the reference genomes, and then an iterative alignment refinement procedure is applied until all the initial pairwise alignments have been fragmented into breakpoint-free segments, which then define a set of marker families.

#### Inferring ancestral adjacencies

An *ancestral adjacency* between two marker families consists of two ancient marker extremities, representing the hypothesis that these two marker extremities are contiguous, in a given orientation, along the unassembled ancient genome of interest, where by contiguous we mean that no other ancient marker is located between them. A *gap* is the sequence between the two marker extremities defining an adjacency. The gap-filling step of AGapEs takes as input a set of potential ancestral adjacencies, each provided with one (or two) template DNA sequence(s) representing a possible hypothesis for the sequence located in the gap between the marker extremities.

We infer such potential ancestral adjacencies using the Dollo parsimony principle: two ancient marker extremities are potentially adjacent if there exist two extant genomes whose evolutionary path contains the most common recent ancestor of the ancient strain of interest and where the two corresponding extant marker extremities are contiguous [see Fig. S7 (available with the online Supplementary Material) for an example].

It follows that every potential ancestral adjacency is supported by a set of extant adjacencies, i.e. adjacencies between extant marker extremities. Therefore, each putative ancestral gap is likewise supported by a set of extant gap sequences.

#### Computing template sequences for ancestral adjacencies

The key element of the approach we describe lies in defining the template sequence, or a set of alternative template sequences, associated to each ancestral gap. We follow again the general parsimony-based approach described in [31], designed for the case of pathogens whose sequence evolve slowly over a historical time-scale. For each ancestral adjacency, we compute a multiple sequence alignment of the supporting extant sequence gaps and apply the Fitch–Hartigan parsimony algorithm [35] to each alignment column to reconstruct a most parsimonious ancestral sequence. If the multiple sequence alignment of extant gaps shows little variation, then a single template sequence can be considered, as we expect that minor variations compared to the true ancestral sequence (substitutions, small indels) will be corrected during the local assembly process outlined below. Alternatively, if larger variations are observed, such as larger indels or a contradicting pattern of presence/absence of an IS in the supporting extant gaps, then alternative templates can be considered, under the hypothesis that the true variant can be recovered from the provided aDNA reads.

#### The gap-filling algorithm

For each given ancestral adjacency, AGapEs aims at filling the gap between the marker extremities using aDNA reads mapped onto the template sequence. Assume we are given a template sequence *t* for the gap associated to an adjacency between two marker extremities of marker families *m_1_* and *m_2_*. Let *a*_1_ and *a*_2_ be the sequences of the ancient genome associated to marker *m*_1_ and marker *m*_2_, respectively, accounting for the orientation implied by the extremities forming the adjacency. We define *R*=*a*_1_+*t*+*a*_2_ as the concatenated nucleotide sequence of the oriented ancient markers and the template.

We first align the aDNA reads with *R* using bwa [36], where only mappings are considered further whose start and/or end position is in *t* (i.e. either fully included in *t* or overlapping the junction between a marker and the gap template). We denote by *M*_*t*_ this set of mappings. Next, we construct a graph *G* where vertices are mappings *m*∈ *M*_*t*_ and there is an edge between two vertices (i.e. mappings) if the two mapping coordinates (segments of *R*) overlap. For each such edge/overlap, we define *s* as the non-overlapping suffix of the mappings with the highest end coordinate. We can then associate a weight to each edge given by the edit distance between *s* and the subsequence *R*_*s*_ of *R* it aligns to. A sequence of overlapping reads that minimizes the distance to *t* can then be found by searching for a shortest path between the vertex labelled with the smallest start position (i.e. the first mapping covering the junction between *a*_1_ and *t*) and the vertex labelled with the largest start position (i.e. the last mapping covering the junction between *t* and *a*_2_). See Fig. S13 for an illustration.

If such a path exists, it can be found with Dijkstra's algorithm [37] implemented based on a min-priority queue in *O(|E|+|V| log |V|)* time, where *V* is the vertex set and *E* the edge-set of *G*. If no such path exists, then there are either regions in *R* that are not covered by any mapped aDNA read or breakpoints in the mapping, where two consecutive bases in the sequence are covered, but not both by the same read. In these cases, uncovered regions and breakpoints need to be identified in the mapping beforehand to identify start and end vertex of the shortest path. We can then obtain a partial gap filling, precisely for the regions covered by mapped reads. If a gap template sequence is only partially covered by mapped aDNA reads, we correct the covered regions as described above and use the template sequence of the uncovered regions to complete filling the gap.

#### Scaffolding ancient genomes

We say that two potential ancestral adjacencies are *conflicting* if they share a common marker extremity, hence, showing some ambiguous scaffolding signal (Fig. S8). Further, ISs are repeats that are known to be involved in genome rearrangement in bacterial pathogens [38] and thus to impact comparative scaffolding, creating potential ambiguity and conflicting adjacencies. We define an *IS-annotated* adjacency as an ancient adjacency that is supported by at least one extant adjacency whose gap contains an IS annotation. An adjacency that is neither conflicting nor IS-annotated is said to be *simple*. We separate all potential ancestral gaps into groups of simple, conflicting and IS-annotated gaps, according to the status of the corresponding adjacency.

For simple and conflicting gaps without IS annotation, we can follow the AGapEs algorithm as described above to fill the gap. For IS-annotated gaps, we reduce the described large variations in the multiple alignment by further dividing its supporting extant gaps into sets of IS-annotated and non-IS-annotated sequences. Building the multiple alignment on each of these sets separately allows us to define two alternative templates that can be used as a basis to fill the gap. Ideally, differences in read coverage or breakpoints naturally identified by AGapEs then point to one of the alternative templates for each IS-annotated gap. See Fig. S12 for an overview.

In order to study genome rearrangements, we generate for each ancient genome a set of *Contiguous Ancestral Regions* (CARs; equivalent for ancient genomes to scaffolds for extant genomes), each defined by a set of ordered markers, with gaps filled as described above. Conflicting adjacencies are related by the marker extremities they share, defining clusters of related conflicting adjacencies. Within such a cluster, each adjacency is supported if the corresponding gap is completely filled by aDNA reads. In order to propose a conflict-free scaffolding, we chose to remove all unsupported conflicting adjacencies. Moreover, if two (or more) conflicting adjacencies share a marker extremity and are supported by aDNA reads, indicating an ambiguity in the support from ancient reads, we remove them all. The set of ancestral adjacencies can then be ordered into CARs. Last, to generate a final assembly, we convert the reconstructed sequences of markers back to genome sequences by filling the gaps with the read sequences if possible and resorting to the template sequence otherwise as described above.

### Data and pre-processing

We now describe the input to our analysis of *Y. pestis*, namely ancient sequencing data, ancient and extant assemblies, and annotations of ISs.

#### Sequencing data

The first aDNA data set was obtained from a London victim of the Black Death pandemic in the 14th century [26] (individual 8291), the second consists of five samples from victims of the Great Plague of Marseille around 400 years later [27]. The aDNA reads were obtained following a capture approach, where probes from extant *Yersinia* genomes are used to design a capture array that allows to enrich DNA from ancient remains for *Yersinia* genomes, followed up by high-throughput DNA sequencing (see [12, 13] for a general description of this approach). As a consequence, ancient genome segments that do not occur in the extant strains used to design the capture arrays are missing in the sequenced aDNA reads.

For the London strain, the array was built using *Y. pestis* strain CO92 together with probes taken from other *Y. pestis* strains to cover 933 SNPs and selected virulence regions; sequencing was then performed on the Illumina Genome Analyzer IIx platform. For the Marseille data set, the array was built also using the CO92 strain, supplemented with additional chromosomal regions from *Y. pestis* biovar Microtus strain 91,001*, Y. pseudotuberculosis* IP 32,953 and *Y. pseudotuberculosis* IP 31,758, with sequencing performed on an Illumina HiSeq 2000 platform (Table S3). The mean read length was 53 bp in the London dataset and 75 bp in the five Marseille samples (Fig. S3).

#### Reference strains and phylogeny

We relied on seven extant *Y. pestis* and four *Y. pseudotuberculosis* as reference and outgroup genomes (see Table S1). The phylogeny of the considered strains is depicted in Fig. S1 and is taken from [26, 27].

#### Contig assembly

We *de novo* assembled aDNA reads into contigs using Minia [39] for both aDNA data sets (London outbreak and Marseille outbreak). Minia is a conservative assembler based on an efficient implementation of the de Bruijn graph methodology. In general, Minia produces shorter contigs than competing assemblers, as it avoids assembly decisions in case of ambiguity in the sequence data. We will refer to the Minia assemblies as *de novo* assemblies in the following.

The Marseille strain data set consists of five samples as described in [27] that we assembled separately with Minia. We first compared the quality of the resulting assemblies by mapping contigs with a fixed minimal length to the genome of the extant strain *Y. pestis* CO92 and summing the total length of the mappings as seen in Fig. S5. While restricting the minimal contig length, two of the samples covered an extensively larger part of the CO92 strain genome and, thus, indicated a better sequencing quality. Fig. S6 shows that if we restrict the minimal contig length, only a small part of the *Y. pestis* reference genomes is covered by contigs from all five samples. We used the assembly of sample OBS116 with a minimal contig length of 500 bp to segment the extant genomes into markers.

#### Insertion sequence annotation

ISs are strongly related to rearrangements in *Y. pestis* evolution, and their annotation in the considered extant genomes is crucial. In order to annotate ISs, we designed our own annotation pipeline. Because IS elements in the original GenBank files were rather disparately annotated, we relied on automated annotations from the Basys annotation server [40]. Basys identified 11 families of IS transposase proteins (see Table S2 and Fig. S2). For each of these families, we produced a multiple alignment of their annotated sequences using muscle [41], which was subsequently used to train Hidden Markov Model (hmm) profiles. Using hmmer [42], we then annotated those regions as associated to IS elements that showed significant correlation to any of the hmm profiles. We eventually combined the GenBank annotations with these derived annotations.

The number of these IS annotations per reference genome ranged from 151 in *Y. pestis* KIM10+ to 293 in *Y. pestis* Antiqua (see Table S1). The length of the annotations ranged from 60 to 2417 bp. Some short annotations deviated from the expected length for ISs; however, in order to avoid filtering any true annotations, we included them all as potential IS coordinates in the downstream analysis.

## Results

### Assembly of the London and Marseille strains

#### Assembling the London strain

The *de novo* assembly of the London data consisted of 4183 contigs of length at least 300 bp that covered 2 631 422 bp (see the Supplementary Material A4). Using the marker segmentation described in the previous section, we subsequently obtained 3691 markers covering 2 215 596 bp in total. Only contigs whose segments aligned uniquely and universally onto the reference genomes are represented in the markers set, explaining the lower number of markers compared to contigs. We obtained 3691 potential ancestral adjacencies: 3483 were simple, 201 were IS-annotated and non-conflicting, and only 7 were conflicting. Among the conflicting adjacencies, five were also IS-annotated, illustrating that most rearrangements in *Y. pestis* that can create ambiguous signal for comparative scaffolding are associated with IS elements (see also Table S5).

For most potential ancestral adjacencies, the lengths of the sequences in extant genomes associated with the supporting extant adjacencies were very similar, indicating well-conserved extant gaps (Fig. S9a, b). There were 21 gaps whose lengths difference fell into the length range of potential annotated IS elements, thus, raising the question of the presence of an IS within these adjacencies in the ancestral genome. We noted a small number of five potential ancestral adjacencies with strikingly large extant gap length differences. All of these gaps accumulated more than one IS annotation in some extant genomes. Most problematic was a gap with a length difference of more than 100 000 bp. As this gap was not well conserved in general (apart from the inserted sequences), it was difficult to obtain a good template sequence based on a very fragmented multiple alignment. We will get back later to this special gap.

**Fig. 1.**
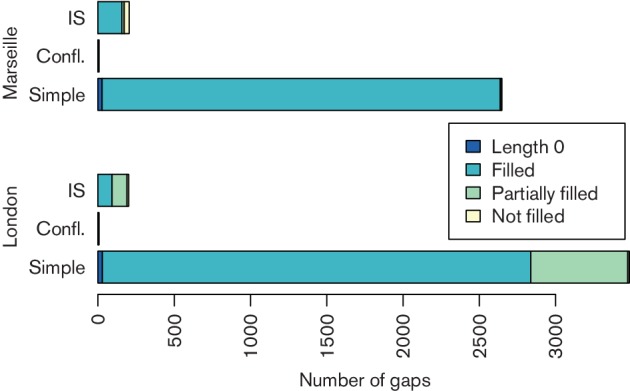
Result of gap filling for both data sets. Note that if a gap is conflicting and IS-annotated, we assigned it to the conflicting group. We differentiated between gaps of length 0 (i.e. both markers are directly adjacent), completely and partially filled gaps, and not filled gaps (Tables S6 and S7).

We applied AGapEs to all potential ancestral gaps. We assumed a gap to be filled, if we found a sequence of reads that covered the whole ancestral gap. For an IS-annotated gap, we considered it filled if only one alternative was covered or if both templates were covered but the IS was only annotated in a single extant genome. In the latter case, we expected the non-IS gap version to be ancestral. If both alternative template sequences were covered, we could not recover the true positive gap at this point and marked it as not filled. Fig. 1 summarizes the gap-filling results (see also Table S5). A large number of gaps was supported by sufficient aDNA read coverage that enabled us to fill the gap with a sequence of overlapping aDNA reads. Especially considering partially covered gaps improved the length of the genome that is supported by reads. Note that we also found covering reads for all gaps of length 0, spanning the breakpoint between directly adjacent markers.

We further computed the edit distance between each reconstructed gap sequence and its previous gap template. For IS-annotated gaps, we computed the distance to a template sequence based on all extant gap occurrences, i.e. without considering alternative templates as described previously. We identified one case where the parsimonious gap sequence based on all extant occurrences of the adjacency excluded the IS. However, if aDNA reads were mapped separately to alternative templates based on IS and non-IS annotated extant gaps, only the IS-annotated gap template was covered.

For IS-annotated gaps, 95 ancestral gaps contained an IS, while 106 ancestral gaps were reconstructed without the IS. From these 95 IS gaps, 22 contained annotations that were shorter than 400 bp; however, they all contained additional longer annotations in the same gap. Analysing the number of ancestral ISs with a Dollo parsimony criterion considering only the extant IS annotations, we had 96 ancestral gaps that contained an IS, indicating a large agreement between the ISs that are conserved by the parsimony criterion and the ISs supported by aDNA reads.

We identified two clusters of conflicting adjacencies (see Fig. S14). One consisted of three adjacencies that were all annotated with IS elements, while the other consisted of four adjacencies, including two IS-annotated adjacencies. In total, only two of these conflicting adjacencies were supported by aDNA reads. All other adjacencies contained uncovered regions indicating potential breakpoints. See Fig. S15 for the read coverage of discarded adjacencies. The resulting assembly contained five CARs.

As mentioned earlier, we observed one gap with highly differing extant gap lengths and very little conservation in the reconstruction. The multiple alignment based on extant gap sequence was very fragmented and the mapping of reads onto this template was poor: the gap contained 211 uncovered regions of 9319 bp in total. See Fig. S16 for an overview of the read coverage for this gap in the *de novo* assembly. As the reconstructed sequence had a high edit distance after partial gap filling, we removed this gap sequence completely at this point to avoid dubious and non-robust reconstructed ancestral sequences.

In addition, we aligned all reads again to the final assembly to assess the amount of uncovered regions in the reconstructed sequences. In total, 88 529 bp are not covered by any read; however, most uncovered regions were rather short (see Figs S19 and S20). Based on this mapping, we ran the assembly polishing tool Pilon [43] on the final assembly. It identified several positions where the assembled base (also present in the template) was the minority in comparison to all reads mapping at this position. As Pilon was not taking the respective bases of the extant genomes into account, it ran the risk of correcting the assembly according to sequencing errors in the reads. In fact, the most frequent proposed substitutions corresponded to the common damage pattern of cytosine deamination observed in aDNA [44]. As a consequence, we only kept small indel corrections by Pilon, but rejected all single-base corrections.

In the improved assembly, 49.88 % of the sequence was based on markers and, hence, directly adopted from the initial assembly. Together with the gaps that have been filled by read sequences, we can say that in total 95.25 % is reconstructed using only the available aDNA reads.

#### Assembling the Marseille strain

We used the assembly of sample OBS116 with a minimal contig length of 500 bp to segment the extant genomes into markers. The assembly consisted of 3089 contigs with a total length of 3 636 663 bp (Fig. S4). The segmentation resulted in 2859 markers with a total length of 3 143 627 bp. We analysed 2859 potential adjacencies: 27 of these gaps had a length of 0, leaving 2832 gaps to fill. Based on the observations above, we joined all sample read sets for filling the gaps in the reconstruction to achieve a better coverage.

We can see in Fig. 1 that with the combined set of reads, we could fill nearly all simple gaps by read sequences. In addition, we obtained a larger number of IS-annotated gaps that were filled in comparison to the London data set. For the IS-annotated gaps, 95 were reconstructed containing the IS, 21 contained IS annotations shorter than 400 bp. Hence, we identified the same number of potential ancestral ISs as for the London strain.

We identified two conflicting components in this set of potential adjacencies (see Fig. S17). Both of them aligned in terms of gap lengths and extant occurrences with the two components observed in the assembly for the London strain. In the first component, again only one conflicting adjacency was covered by reads. However, this was a different adjacency in comparison to the London strain, while we had no read support for the gap that was covered in the London data set. This could indicate a potential point of genome rearrangement (see Discussion). In the second component, all involved adjacencies were covered by reads from the five samples. In order to obtain a set of high confidence ancestral CARs, we removed all conflicting adjacencies in this component from the set of potential adjacencies. The coverage of all discarded adjacencies is shown in Fig. S18.

This resulted in six CARs for the ancestral genome. Again, we used bwa [36] to align reads from all five samples to the assembly to assess the amount of uncovered regions in the reconstructed sequences. In total, only 54 672 bp in this mapping were not covered by any read and the length of the uncovered regions was rather short (see Fig. S20).

#### Comparison of the London and Marseille strains

**Fig. 2.**
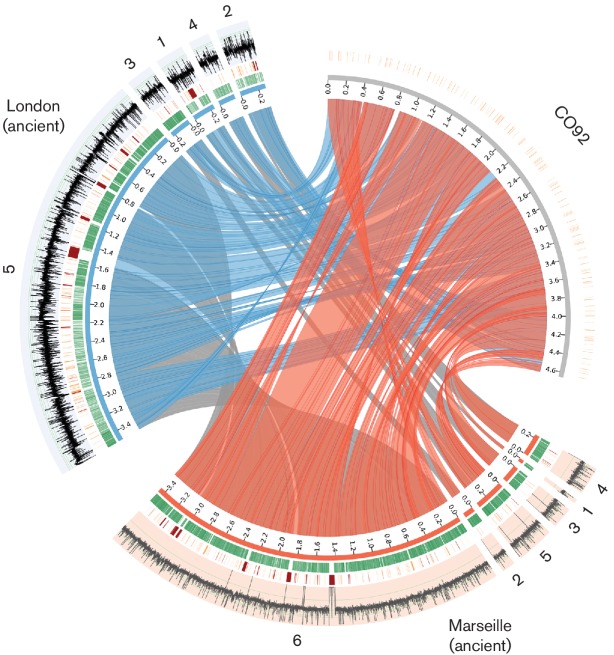
Comparison between the *de novo* assembly of the London strain (blue) and the Marseille strain (red) with the reference *Y. pestis* CO92. The inner links connect corresponding CARs in the reconstructions and the reference. Note that there is only a small inversion, marked in black among the grey links. The positions in both reconstructions covered by markers are indicated in green. All gaps that have IS annotations in the extant genomes are shown in orange. For CO92, all IS annotations are shown as well. In addition, gaps that are only partially filled or have very unconserved extant gap lengths are indicated in red. Finally, the outermost ring shows the mean read coverage in windows of length 200 bp in log scale. The figure was made with Circos [57].

As the Marseille *Y. pestis* strain is assumed to be a direct descendant of the London strain [27], we aligned the obtained CARs in both reconstructions to identify genome rearrangements. As shown in Fig. 2, apart from one larger deletion and one larger insertion in the Marseille strain related to the removed gap sequence in the London strain and a small inversion of length 4138 bp (marked in black), the reconstructed CARs showed no larger rearrangements between both genomes (grey links). The difference in conflicting adjacencies kept is a possible indication for a rearrangement that, however, cannot be explicitly identified at this point. It causes the split pattern observed between CAR3 and CAR1 in the London strain, and CAR2 and CAR5 in the Marseille strain. Given that the available read data did not allow us to further order the resulting CARs into a single scaffold, additional potential rearrangements could be assumed to be outside of the reconstructed CARs. In contrast, Fig. 2 depicts several inversions and translocations between both ancient sets of CARs and the extant *Y. pestis* CO92 (red and blue links, respectively).

To clarify this further, we computed all potential orderings for both sets of CARs and determined the Double-Cut-and-Join (DCJ) [45] genome rearrangement distance (see Supplementary Material A11) for all such orderings between both ancient strains as well as in comparison to *Y. pestis* CO92. We obtained a weighted mean distance of 4.04 between both ancient strains and a mean distance of 11.16 to CO92, with a sd of 0.89 and 0.83, respectively. This suggests a much slower evolution in terms of rearrangements between both ancient strains and the extant strain.

#### Evaluation

##### Influence of initial assembly

Bos *et al*. [26] described a reference-based assembly of the London strain consisting of 2134 contigs of length at least 500 bp. It was obtained with the assembler Velvet [46] using the extant strain *Y. pestis* CO92 as a reference. In order to assess the influence of the reference sequence in the assembly of the ancient genome, we ran our pipeline using this initial assembly to compare to our results based on the *de novo* assembly (see Table S4 and Figs S10 and S11).

We compared the two sets of CARs obtained from both initial assemblies by aligning the resulting genome sequences using MUMmer [47]. We observe no rearrangements between both resulting sets of CARs (see also Fig. S19 and Table S8), showing that, in terms of large-scale genome organization, the final result does not depend on the initial contig assembly.

##### Assembly comparison

We compared our results to assemblies obtained with several other assembly pipelines. We used the *iMetAMOS* pipeline [48] to determine the best *de novo* assembly for both data sets testing different assemblers. The winning assembly computed by SPAdes [49] for both data sets, as well as the Minia assemblies on both datasets, were subsequently used as input for two comparative scaffolding programs, Ragout [32] and MeDuSa [33], to obtain a scaffolding of the initial contigs considering the extant reference genomes in a phylogenetic context. For all scaffolds, we ran Gap2Seq [50] to close the gaps. We will distinguish the results according to the scaffolding tool used in the following.

As shown in Table 1, for both datasets, Ragout reconstructed the smallest number of contigs; however, the scaffolds still contained a high number of unfilled gaps that could not be closed by Gap2Seq. See Table S10 for the results of all tool combinations and Tables S11 and S12 for gene predictions. Our AGapEs reconstruction – although slightly more fragmented – achieved the best assembly likelihood according to both the lap [51] and cgal [52] score. The MeDuSa scaffolder was not able to estimate the gap sizes needed as input for Gap2Seq. Hence, the better likelihood in comparison to Ragout can be accounted to the missing gaps characterized by Ns in the Ragout assembly. Also worth noting with the Marseille strain, MeDuSa was not able to correct a larger than expected contig assembly obtained with SPAdes. Finally, the Minia-AGapEs assemblies did not contain Ns due to the filling of the gaps uncovered by reads by the template sequence, we have indicated the length of template sequence used in parentheses instead.

**Table 1.** Assembly statistics for both data sets, based on contigs with a minimal length of 500 bp. All program parameters are given in Table S9 The lap and cgal likelihoods have been computed based on all reads mapping to any of the reference sequences. Ragout and MeDuSa depend on the quality of the initial assembly in terms of assembled sequence length; hence, we omit results for the Minia assembly here and refer to Table S10.

Strain	Assembly	No. of contigs	Total length (bp)	No of Ns	N50	LAP	CGAL
**London**	SPAdes	2555	3 792 691	0	1888	−11.01048	−6.90196e+08
	Minia	4183	2 631 422	0	930	−15.69016	−7.98656e+08
	SPAdes-Ragout	1	4 068 385	776 139	–	−12.52232	−4.8192e+08
	SPAdes-MeDuSa	77	4 333 801	1917	700 415	−7.97066	−5.00106e+08
	Minia-AGapEs	5	4 441,104	0 (313 628)	3 511 710	−7.26576	−3.55155e+08
**Marseille**	SPAdes	3201	6 072 375	0	4592	−11.03336	−6.0411e+08
	Minia	3089	3 636 663	0	1368	−15.05058	−8.71446e+08
	SPAdes-Ragout	2	4 564 323	542 013	4 530 296	−13.34526	−5.84186e+08
	SPAdes-MeDuSa	2155	6 052 372	618	1 643 585	−10.88342	−6.12532e+08
	Minia-AGapEs	6	4 350 872	0 (184 003)	3 459 919	−8.05526	−4.32647e+08

##### IS reconstruction

In order to validate the IS reconstruction in our assemblies, we ran the tool ISseeker [53] that allows annotation of IS elements in draft genome assemblies by blasting flanking sequences against a reference. We tested both SPAdes and Minia assemblies for the presence of 10 *Y. pestis* species-specific IS elements found in the ISFinder database [54] and using all potential IS gaps as references.

While ISseeker was not able to annotate IS elements in the Minia assembly, in Table 2 we note 30 annotations that are found in the SPAdes assembly. Seven of these were not annotated in the AGapEs reconstruction, and they all concern gaps that are only partially covered by reads. However, a manual check of these gaps determined the presence of the respective IS element in five gaps, indicating that ISseeker was not able to correctly annotate these elements in these cases.

**Table 2.** IS annotations in the London dataset identified by ISseeker in either draft assembly, AGapEs reconstruction or both

	SPAdes	AGapEs	SPAdes and AGapEs	Minia	AGapEs	Minia and AGapEs
IS gap	7	55	23	0	78	0

##### Simulations

We further evaluated AGapEs on simulated data by removing *Y. pestis* CO92 from the set of considered references and simulating six aDNA single-end read datasets with gargammel [55] based on this genome sequence with a mean read length of 60 bp. To test factors influencing the initial assembly of the aDNA reads, we varied the mean coverage of reads from 20× to 10× and in addition simulated different rates of bacterial contamination from 0 to 40 % as provided by the gargammel software. We assembled the simulated reads with Minia [39], setting *k*=19, and subsequently used all contigs with a minimal length of 300 bp as input to our pipeline, considering all adjacencies conserved at the ancestor of *Y. pestis* Z176003 and Antiqua (see Fig. S1).

**Fig. 3.**
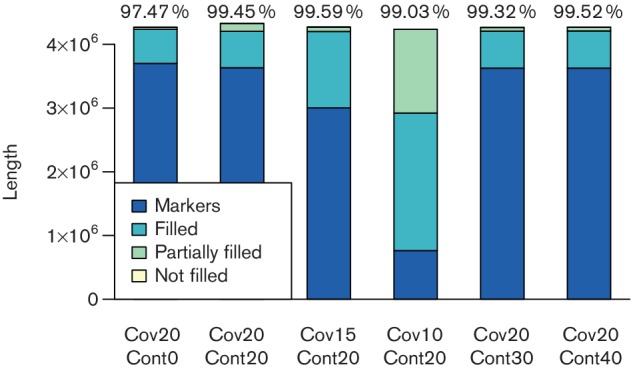
Length of reconstructed sequence defined by markers, completely filled gaps, partially filled gaps (only the covered parts are considered) and unfilled gaps. The simulation parameters vary in terms of read coverage (cov) and simulated bacterial contamination (cont). Above each bar, the percentage of the reconstructed sequence supported by the aDNA reads is given.

For all sets of parameters indicated in Fig. 3, we had a single conflicting component consisting of three adjacencies. Since all are covered by reads, we removed them from the reconstruction, resulting in eight CARs for each simulated experiment. There were no rearrangements between all six reconstructed sequences, they only differ by the ratio of sequence defined by markers, filled and unfilled gaps as depicted in Fig. 3.

The results show that both a reduced read coverage, as well as an increased contamination in the sequencing data, can be handled by our method. The dataset with 10× coverage already shows a reduced marker coverage due to the quality of the assembly, and a further reduced coverage will likely result in missed rearrangement events hidden in very large gaps between markers. The datasets with simulated bacterial contamination show that our method can handle contamination, since contigs are aligned to reference sequences to obtain markers, hence, higher rates of contamination are filtered out. However, it should be noted that assembly software not specifically designed to handle metagenomic datasets will likely not produce a good quality assembly sufficient to compute markers for reconstruction.

## Discussion

In this paper, we have presented a method to fill the gaps between contigs assembled from aDNA reads that combines comparative scaffolding using related extant genomes and direct aDNA sequencing data, and we have applied it to two ancient *Y. pestis* strains isolated from the remains of victims of the second plague pandemic. Although initially designed for the analysis of ancient *Y. pestis* strains, the method implemented in AGapEs is quite generic and can in principle be applied to arbitrary ancient pathogens for which appropriate data is available. More precisely, AGapEs has been designed to work better with aDNA sequencing data with good coverage of the considered ancient genome and available related extant genomes that can be used to generate template sequences for the assembly gaps to be filled. Indeed, in the absence of reliable template sequences or ancient reads for large parts of the ancient genome, AGapEs will result in large unfilled or partially filled gaps that will improve the initial contig assembly, but leave open ambiguities in terms of scaffolding and ancient genome sequence. For example, a challenging data set for AGapEs would be the data set obtained from plague victims in Ellwangen [56], which is characterized by the fact the sequenced aDNA reads are expected to cover only three quarters of the ancient genome. However, AGapEs is robust to an initially very sparse contigs assembly, as illustrated by the low 10× depth-of-coverage (DoC) simulated data set, where we observed that a very low coverage by contigs-based markers resulted into 99 % of the improved assembly covered by ancient reads, although a large number of gaps is only partially filled, a feature that does not manifest with a simulated 15× DoC data set. On real data, the comparison of the two assemblies for the London strain illustrates also that relying on a shorter initial *de novo* contig assembly does not impact significantly the final result. The results we obtained with the Marseille data set illustrate that if a good coverage of reads over the whole genome can be provided (as through multiple sequencing experiments for multiple samples), even a cautious initial contig assembly can be improved in such a way that most gaps are filled using unassembled aDNA reads. With both data sets, we obtained largely improved genome assemblies, with a reduced fragmentation (from thousands of contigs to a handful of CARs) and a very small fraction of the final assembly that is not supported by aDNA reads.

Applied to the same data set for the London strain, the method FPSAC [31] was able to obtain a single scaffold based on parsimonious optimization. Comparing our resulting assembly to this single scaffold, we could identify two breakpoints between both assemblies; hence, both methods did not entirely support the same scaffold structure for the London strain. These disagreements should be seen as weak points in both assemblies, as they were not reconstructed by different scaffolding objectives and would need to be confirmed more confidently by additional sequencing data.

We see a clear connection between conflicts in the set of potential adjacencies and the presence of IS elements in the corresponding gaps. Solving these conflicts based on aDNA read data provides a useful way to identify ancestral adjacencies in a conflicting component if the quality of the aDNA data is sufficient. The mapping of aDNA reads has shown to be mostly difficult at repetitive regions like ISs, where the presence of the IS in the ancestral gap cannot be clearly detected by the aDNA sequencing data.

Interestingly, the improved assemblies of the London and Marseille strains showed no explicit large genome rearrangements except for a small inversion. Even if potential genome rearrangement might not be observed due to the fragmentation of the assemblies into CARs, the synteny conservation between two strains separated by roughly 400 years of evolution is striking compared to the level of syntenic divergence with extant strains. This might be explained by the fact that both the London and Marseille strains belong to a relatively localized, although long-lasting, pandemic [27]. Also of interest is the observation that conflicting adjacencies in the Marseille data set were covered by aDNA reads, thus, making it difficult to infer robust scaffolding adjacencies. This raises the question of the presence of several strains in the Marseille pandemic that might have differed by one or a few inversions. It is also important to recall that both the London and Marseille strains data were obtained through an aDNA capture protocol that uses DNA baits from extant strains. This data acquisition protocol, together with a comparative approach for scaffolding and gap filling, will not recover any ancient genome sequence that has not been conserved in the modern strain genomes. Recovering such genome segments that have disappeared during evolution is an important question for which new methods are needed.

Answering these questions with confidence would require additional targeted sequencing of a few regions of the genomes of the London and Marseille strains, or the sequencing, with sufficient coverage, of additional strains involved in the second plague pandemic from outbreaks that occurred at various time points. Nevertheless, it is likely that progress in aDNA sequencing technologies and protocols, coupled with methods such as AGapEs to scaffold and fill gaps in contigs assemblies, will soon lead to a better understanding of the evolution of genome organization in *Y. pestis* during the second plague pandemic.

## Data bibliography

1. Bos KI, Schuenemann VJ, Golding GB, Burbano HA, Waglechner N *et al.* NCBI SRA SRX096047 (2013).
2. Bos KI, Herbig A, Sahl J, Waglechner N, Fourment M *et al.* ENA PRJEB12163 (2016).
3. Rosso ML, Chauvaux S, Dessein R, Laurans C, Frangeul L. *et al.* NCBI RefSeq NC_003143.1 (2008).
4. Chain PS, Hu P, Malfatti SA, Radnedge L, Larimer F. *et al.* NCBI RefSeq NC_008150.1 (2006).
5. Shen X, Wang Q, Xia L, Zhu X, Zhang Z. *et al.* NCBI RefSeq NC_014029.1 (2010).
6. Chain PS, Hu P, Malfatti SA, Radnedge L, Larimer F. *et al.* NCBI RefSeq NC_008149.1 (2006).
7. Deng W, Burland V, Plunkett G III, Boutin A, Mayhew GF. *et al.* NCBI RefSeq NC_004088.1 (2002).
8. Song Y, Tong Z, Wang L, Han Y, Zhang J. *et al.* NCBI RefSeq NC_005810.1 (2003).
9. Copeland A, Lucas S, Lapidus A, Barry K, Detter JC. *et al.* NCBI RefSeq NC_009381.1 (2007).
10. Eppinger M, Rosovitz MJ, Fricke WF, Rasko DA, Kokorina G. *et al.* NCBI RefSeq NC_009708.1 (2007).
11. Challacombe JF, Bruce D, Detter JC, Green L, Land M. *et al.* NCBI RefSeq NC_010465.1 (2008).
12. Challacombe JF, Bruce D, Detter JC, Green L, Land M. *et al.* NCBI RefSeq NC_010634.1 (2008).
13. Chain PS, Carniel E, Larimer FW, Lamerdin J, Stoutland PO. *et al.* NCBI RefSeq NC_006155.1 (2004).

## Supplementary Data

3437 Supplementary File 1
