# Supplementary material for: Comparative scaffolding and gap filling of ancient bacterial genomes applied to two ancient Yersinia pestis genomes
Source: Microb Genom. 2017 Jul 8;3(9):e000123. doi: 10.1099/mgen.0.000123 (PMC5643016; doi:10.1099/mgen.0.000123)
Supplement: Supplementary File 1 [file mgen-3-123-s001.pdf]

A Supplementary Material

A.1 Reference genomes

Table S1. Accession numbers of full assemblies for the chromosome of all extant *Yersinia pestis* and *Yersinia pseudotuberculosis* reference genomes. In addition, the number of IS annotations per reference is given.

Strain	Accession no.	IS annotations
Yersinia pestis		
CO92	NC_003143.1	233
Antiqua	NC_008150.1	293
Z176003	NC_014029.1	170
Nepal516	NC_008149.1	212
KIM10+	NC_004088.1	151
biovar Microtus 91001	NC_005810.1	168
Pestoides F	NC_009381.1	190
Yersinia pseudotub.		
IP 31758	NC_009708.1	-
YPIII	NC_010465.1	-
PB1/+	NC_010634.1	-
IP32953	NC_006155.1	-

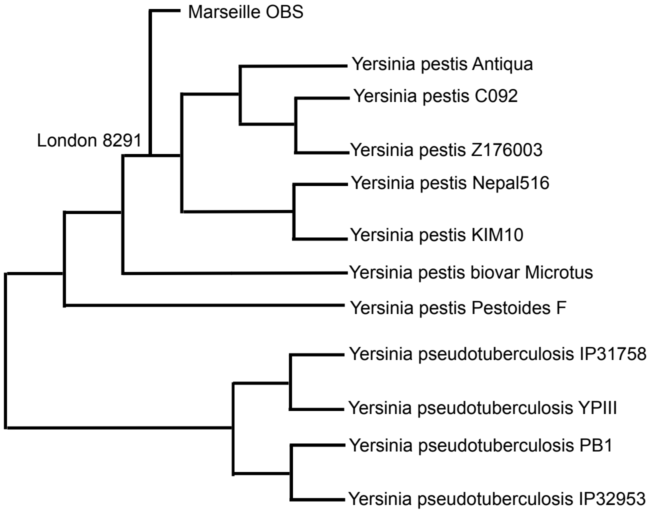

Figure S1. Yersinia pestis phylogeny, including seven extant *Yersinia pestis* strains, four extant *Yersinia pseudotuberculosis* strains and two ancient *Yersinia pestis* strains from the London and Marseille outbreak of the bubonic plague.

Table S2. IS families obtained from BASys annotations.

IS family	Number of sequences
IS1328	6
IS150	71
IS200	568
IS21	8
IS257	13
IS3A	12
IS4	2
IS5376	343
IS911	15
IS911B	15
ISRM3	243

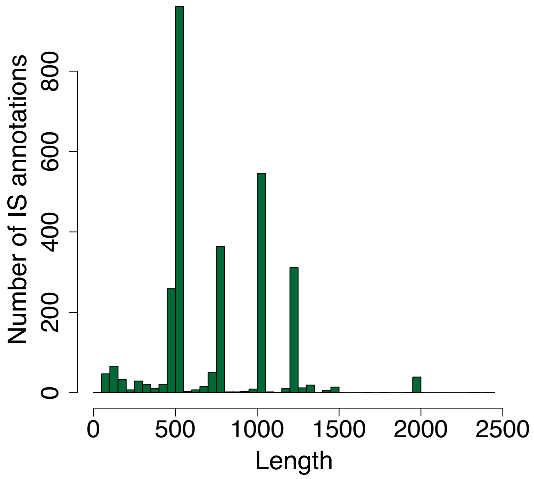

Figure S2. Lengths of all potential IS annotations in all reference genomes.

A.2 Ancient read data

The read set for the London strain individual 8291 (Genbank accession SRA045745) consists of merged single-end reads obtained by array-based enrichment and Illumina sequencing[3]. The read set for the Marseille strain (European Nucleotide Archive under accession PRJEB12163) consists of five samples obtained by array-based enrichment and Illumina sequencing as well[2]. For this data set, we merged the paired-end reads according to the preprocessing described in the next section.

Table S3. Read classification with kraken[13] against a local copy of NCBI Nucleotide Database to obtain a taxonomic classification for reads in both datasets. The table contains an extract of the report computed by kraken, with the two most frequent genera in addition to the Yersinia classification.

London			Marseille		
Taxon	# reads	% of reads	Taxon	# reads	% of reads
unclassified	2672978	27.21	unclassified	9,825,271	58.31
Bacteria	7138248	72.67	Bacteria	6,856,243	40.69
Yersinia	3772442	38.40	Yersinia	4,550,983	27.01
Y. pestis	375138	3.82	Y. pestis	726615	4.31
Y. pseudotuberculosis	3127	0.03	Y. pseudotuberculosis	7462	0.04
Y. enterocolitica	1574	0.02	Y. enterocolitica	2337	0.01
Rhodanobacter	9302	0.09	Mycoplasma	21049	0.12
Pusillimonas	8207	0.08	Burkholderia	12697	0.08

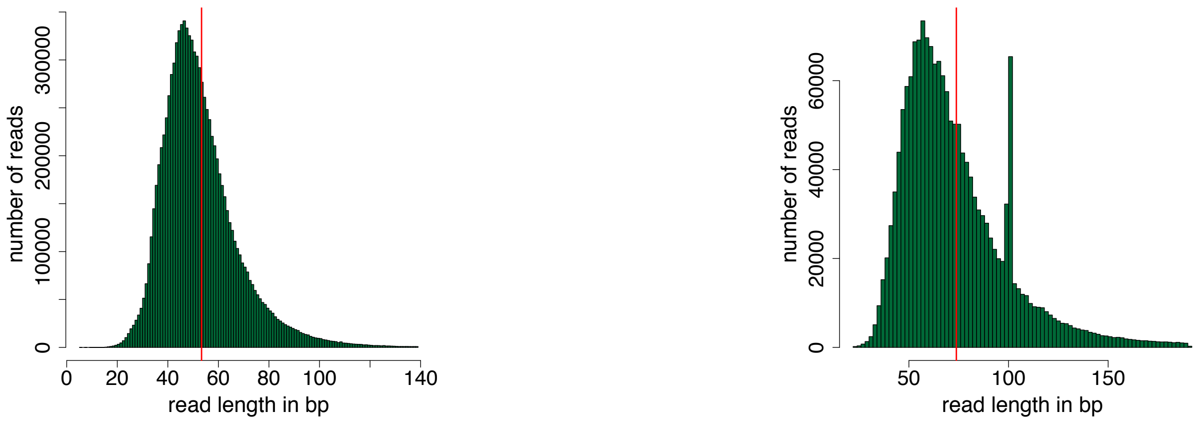

Figure S3. Read length distribution for reads in datasets 8291 (London) and OBS116 (Marseille).

A.3 Preprocessing of reads

The raw reads of all OBS samples have been preprocessed and merged (as described in[2]):

```
1) Trim adapters separately for R1 and R1, adapter sequence given
# -a adapter sequence
# -e maximum allowed error rate (no. of errors divided by the length
# of the matching region)
# -O minimum overlap length between read and adapter
cutadapt -a AGATCGGAAGAGC -e 0.16 -O 1 $READS_R1 -o "${SAMPLE}_R1t.fastq"
cutadapt -a AGATCGGAAGAGC -e 0.16 -O 1 $READS_R2 -o "${SAMPLE}_R2t.fastq"
3) Merge trimmed reads
# -m minimum required overlap length between two reads to provide a
# confident overlap
# -x maximum allowed ratio between the number of mismatched base pairs
# and the overlap length
flash "${SAMPLE}_R1t.fastq" "${SAMPLE}_R2t.fastq" -m 11 -x 0.15
-o "${SAMPLE}_R12t.fastq"
4) Concatenate merged reads and unmerged R1s and filter out reads
shorter than 24 bases
./min_length.py "${SAMPLE}_R121.fastq" "${SAMPLE}_R121_24.fastq"
```

A.4 Contig assembly

For both datasets, we assembled aDNA reads with minia[4] with different values of the k-mer threshold $k \in \{17, 19, 21\}$ and a minimal k-mer occurence of 3. We evaluated the total contigs length with regards to a minimal contig length threshold $\in \{200, 300, 400, 500\}$. The total contig length can indicate how much of the expected genome size the assembled contigs can cover, while a higher minimal contig threshold can provide a better base for defining markers. We found the best trade-off with $k = 19$ and a minimal contig length of $300bp$ for the 8291 dataset and $k = 21$ and a minimal contig length of $300bp$ for the OBS116 dataset. In addition, Bos et al[3] describe a reference-based assembly of the London strain consisting of 2,134 contigs of length at least 500bp. It was obtained with the assembler Velvet[14] using the extant strain *Yersinia pestis CO92* as a reference. In order to assess the influence of the reference sequence in the assembly of the ancient genome, we compare our pipeline using this initial assembly to our results based on the de novo assembly. We will refer to the assembly by Bos et al. as *reference-based* in the following. As expected, the de novo assembly is more fragmented with 4,183 contigs of length at least 300bp that cover 2,631,422 bp. We compared both contig assemblies by aligning them with MUMmer[7]. Unaligned bases mostly belong to regions in the reference-based assembly that have not been assembled in the conservative de novo assembly, and only an extremely low amount of nucleotide variations can be observed (Table S4), and no observed genome rearrangement.

Table S4. Comparison of contigs in reference-based and de novo assembly of the London data set.

	reference-based assembly Velvet[3, 14]	de novo assembly minia[4]
Length threshold L	500 bp	300 bp
Number of contigs $> L$	2134	4183
Total contig length	4,013,159 bp	2,631,422 bp
Aligned contigs	1,866 (87.44%)	3,885 (92.88%)
Aligned bases	2,414,881(60.17%)	2,380,757(90.47%)
Unaligned bases	1,598,278 (39.83%)	250,665 (9.53%)
Single Nucleotide InDels	14	
SNPs	39	

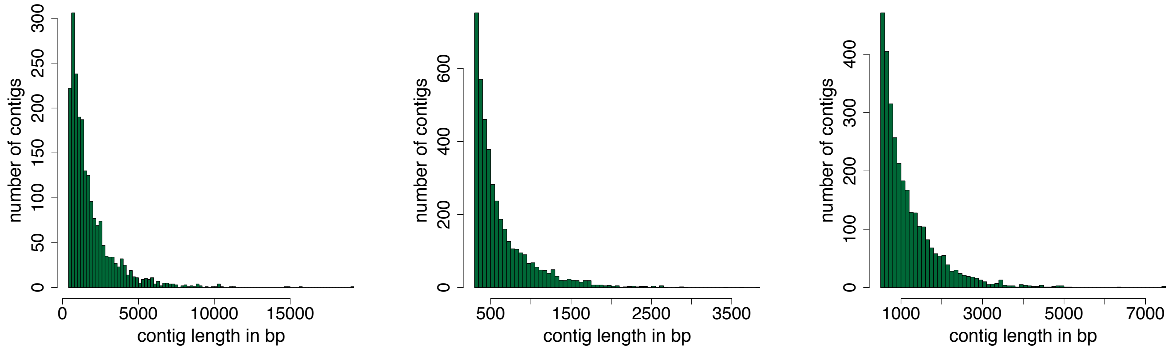

Figure S4. Contig length distribution for (1) all contigs longer than 500bp in the reference-based assembly for the London dataset and (2) all contigs longer than 300bp in the de novo assembly for the London dataset and (3) all contigs longer than 500bp in the de novo assembly for Marseille dataset OBS116.

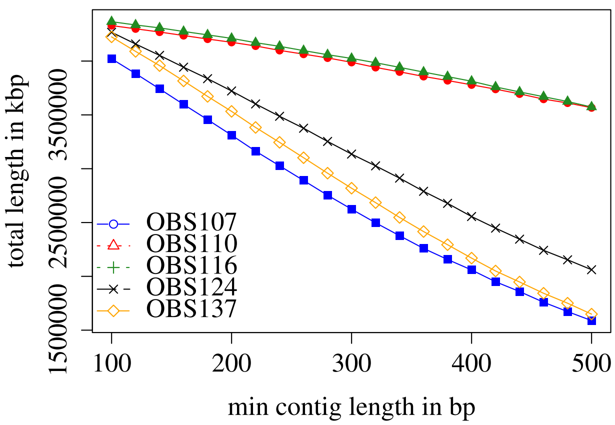

Figure S5. Total length of contigs mapped to *Yersinia pestis CO92* greater than a minimum contig length for Marseille samples.

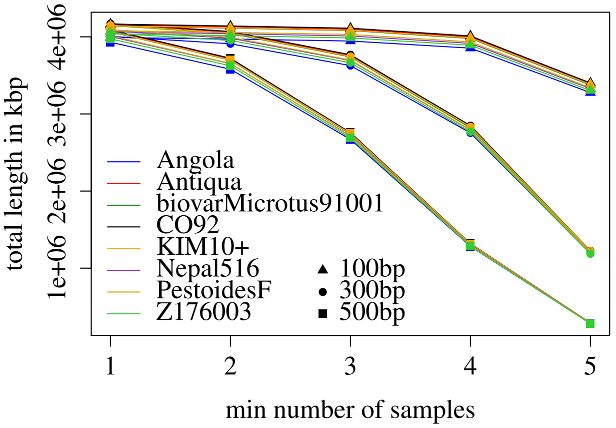

Figure S6. Comparison of contigs by mapping to different reference sequences. While most of the references are covered by at least one sample, only a small part of the references is covered by all Marseille samples.

A.5 Ancestral marker adjacencies

We define ancestral markers as described in[10]. Potential ancestral adjacencies are defined according to the Dollo parsimony criteria. We obtain 2,207 markers that cover 3,463,281 bp in total for the reference-based assembly. For the de novo assembly, we obtain 3,691 markers covering 2,215,596 bp in total. All markers for the de novo assembly are contained in or overlapping with markers from the reference-based assembly.

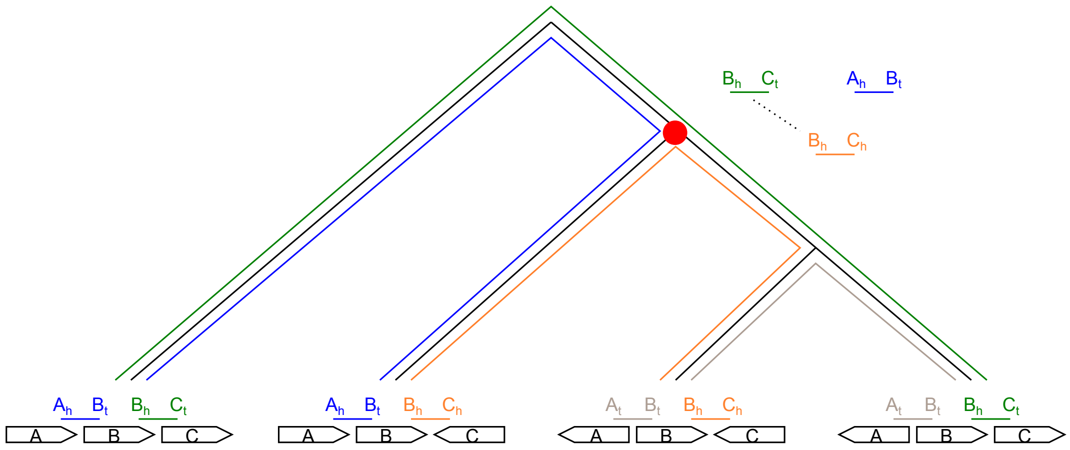

Figure S7. Defining the set of potential ancestral adjacencies, given extant adjacencies at the leaves of the phylogeny. An adjacency is potentially ancestral if it is present in two extant leaves whose path in the tree contains the ancestor of interest (red dot). In this example, the blue, green and orange adjacencies fulfill this criterion, while the grey adjacency is not potentially ancestral. Note that the set of potential ancestral adjacencies is not consistent: The green and orange adjacencies are conflicting as they share the same marker extremity B_h (see also Figure S8.)

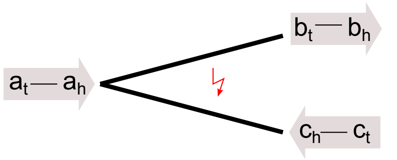

Figure S8. Example for conflicting adjacencies. Each marker a,b and c is depicted by its extremities in order to decode its orientation in the genome. The two adjacencies $\{a_h, b_t\}$ and $\{a_h, c_h\}$ are conflicting and cannot be part of a consistent reconstructed genome in the same time. See also S14 and S17 for conflicting adjacencies in both ancient *Yersinia pestis* datasets.

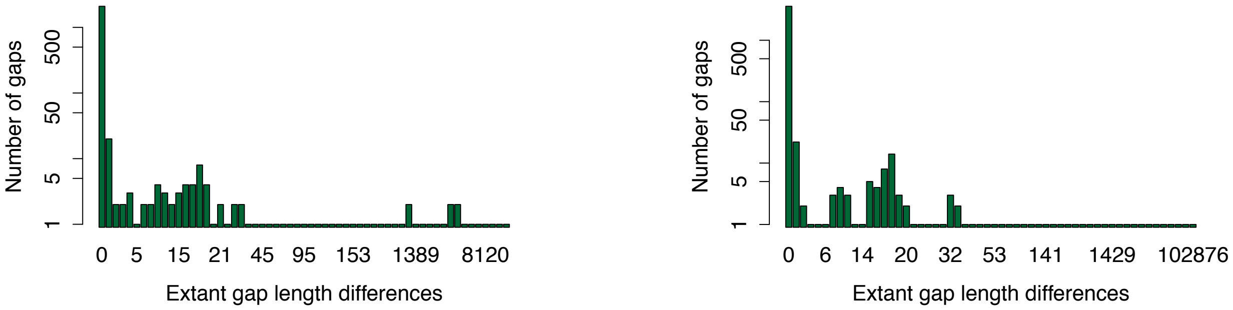

Figure S9. Length variation of extant gaps supporting potential ancestral adjacencies for the 8291 dataset: (a) for markers based on the reference-based assembly, (b) for markers based on the de novo assembly.

A.6 Read mapping

For mapping the reads to the template gap sequences, we used *BWA*[8] with parameter -a to keep all alignments for each read and samtools rmdup to remove PCR-duplicates. In order to correctly identify breakpoints in the mappings, we also removed all clipped alignments.

```
bwa mem -a  ${GAP} ${READS}.fastq > ${GAP}.sam
awk '$6 !~ /H|S/{print}' ${GAP}.sam | samtools view -S -F 4 -b > ${GAP}.bam
samtools sort ${GAP}.bam ${GAP}_mapped
samtools rmdup -s ${GAP}_mapped.bam ${GAP}_mapped_d.bam
```

A.7 Filling of gaps between adjacent markers

For the reference-based assembly, we inferred 2,208 potential adjacencies: 1,991 are simple, 207 IS-annotated but non-conflicting, and 10 are conflicting. Among the conflicting adjacencies 8 are also IS-annotated, illustrating that most rearrangements in *Yersinia pestis* that can create ambiguous signal for comparative scaffolding, are associated with IS elements. We have 28 and 21 gaps in the reference-based and de novo assembly respectively whose lengths difference falls into the length range of potential annotated IS elements, thus raising the question of the presence of an IS within these adjacencies in the ancestral genome. We note a small number of potential ancestral adjacencies with strikingly large extant gap length differences (7 and 5 in the respective assemblies).

For each filled gap, we computed the edit distance between the read-based gap sequence and the respective template for both assemblies (see Figure S11). Especially for IS-annotated gaps, this allows us to compare the filled gap sequence with the reconstructed gap sequence if IS annotations are ignored. One gap annotated with an IS elements in the reference-based assembly shows a larger distance of 1959 to the template, corresponding to the annotated length of the IS. While the template does not include the IS sequence, the mappings of the reads shows clear breakpoints at the respective gap for the non-IS template and provides full coverage for the IS template.

Table S5. Results of gap filling for both assemblies of the London dataset. If a gap is conflicting and IS-annotated, we assign it to the conflicting group.

	reference-based assembly			de novo assembly		
	consistent	conflicting	IS	consistent	conflicting	IS
gaps of length 0		48			29	
gaps filled	1,162	2	109	2808	2	92
length (bp)	172,614	7,876	70,550	710,138		86,805
gaps partially filled	718	-	84	637	-	98
total length (bp)	319,633	-	240,085	862,307	-	505,856
coverage by reads (bp)	245,779	-	194,414	765,406	-	443,090
gaps not filled	63	8	14	9	5	11
length (bp)	7,154		172,689	25,777		18,249
total number of gaps	1,943	10	207	3454	7	201
total gap length (bp)	499,401		483,324	1,598,222		610,910
total assembly length	4,398,214			4,441,004		
coverage by marker	3,463,281 (78,74 %)			2,215,596 (49.88 %)		
coverage by reads	4,154,514 (94.46 %)			4,230,162 (95.25 %)		

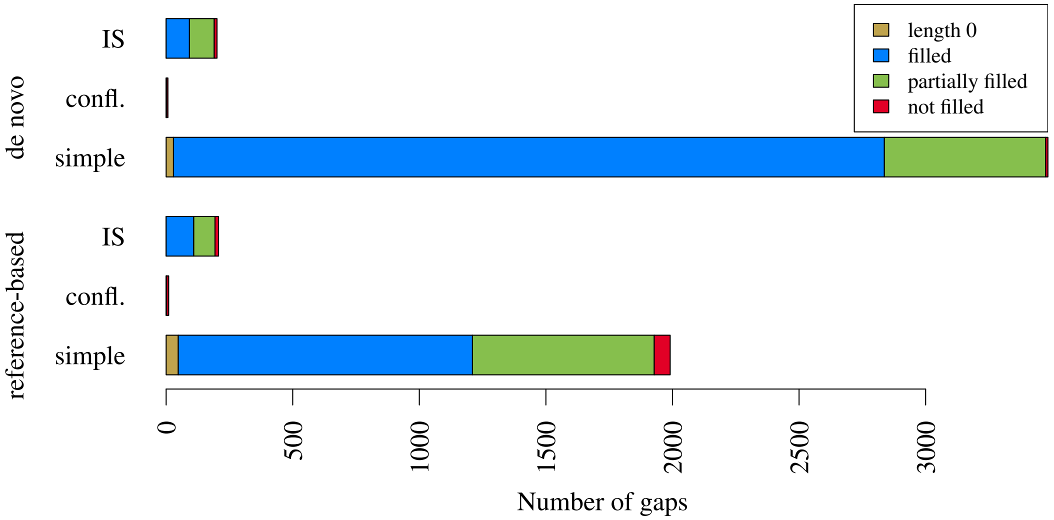

Figure S10. Result of gap filling for the reference-based as well as the de novo assembly for the London data set. Note that if a gap is conflicting and IS-annotated, we assign it to the conflicting group. We differentiate between gaps of length 0 (i. e. both markers are directly adjacent), completely and partially filled gaps and not filled gaps.

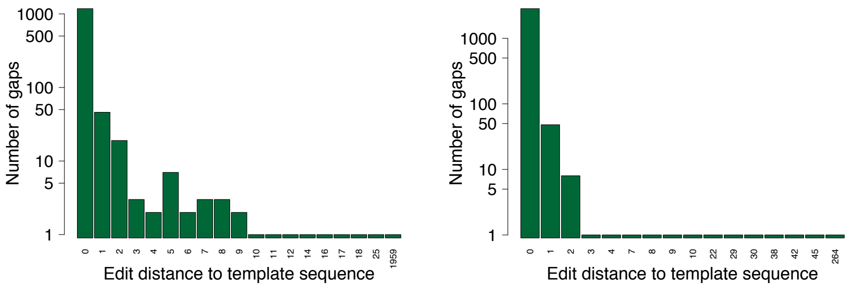

Figure S11. (a) Edit distance between reconstructed gap sequence and template sequence for all covered gaps in the reconstruction for (a) the reference-based assembly, (b) the de novo assembly.

Table S6. Gaps filled in the marseille dataset.

	consistent	conflicting	IS
gaps of length 0		27	
gaps filled	2610	4	157
length (bp)	751634	13231	222079
gaps partially filled	9	-	15
total length (bp)	15001	-	34223
covered by reads (bp)	6140	-	28650
gaps not filled	1	3	33
length (bp)	130		77125
total assembly length		4,350,872	
covered by markers		3,143,627 (72.25 %)	
covered by reads		4,165,361 (95.73 %)	

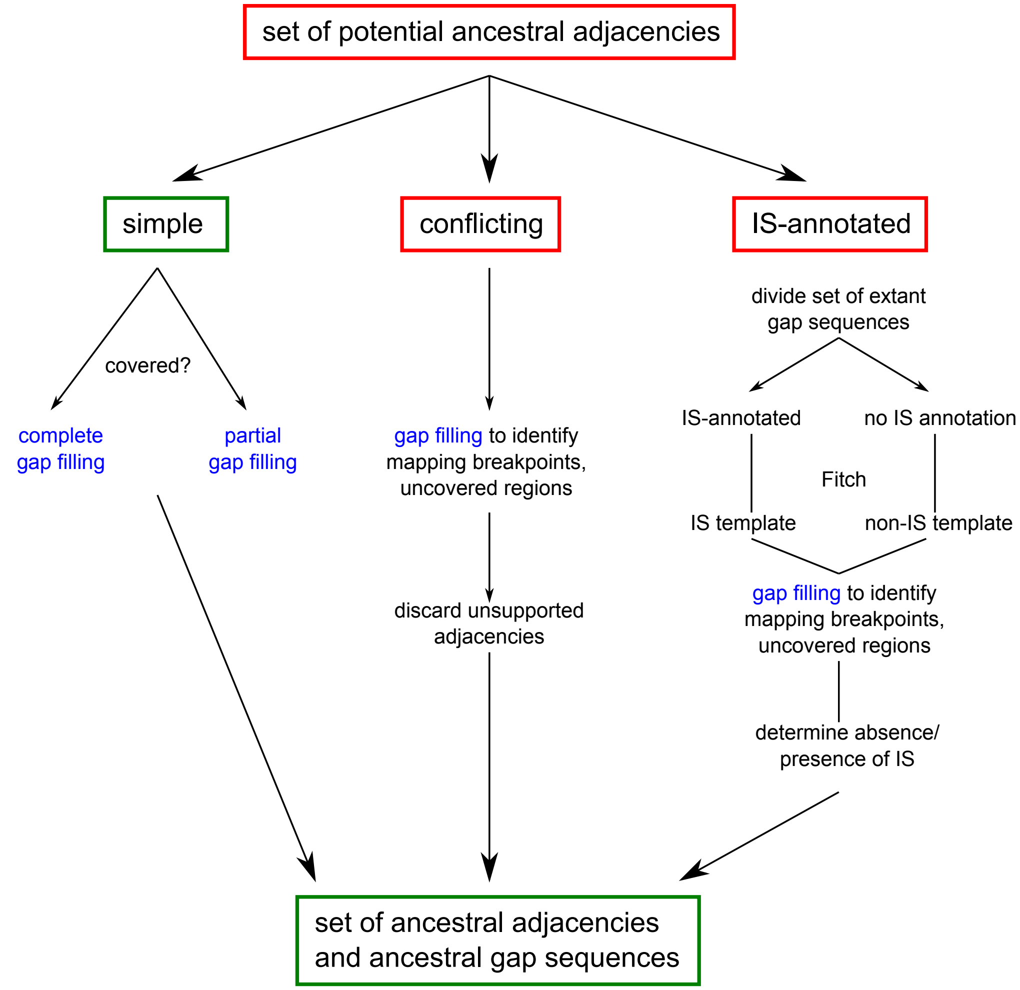

Figure S12. Overview over the pipeline based on the AGapEs method applied to all ancient data sets in this paper.

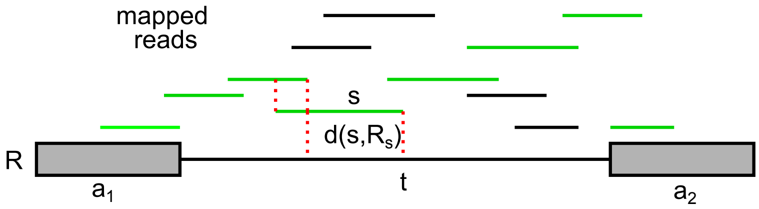

Figure S13. Example for a mapping of ancient reads to an adjacency and a template gap sequence. The overlapping set of reads depicted in green covers the gap template. The overlap indicated in red is represented by one edge in the graph constructed by AGapEs, the non-overlapping suffix of the mapping is considered to weight the edge by the edit distance.

A.8 Comparison with gap2Seq

The gap2Seq algorithm aims at closing gaps in assemblies as an exact path length problem on a de-bruijn graph of the given reads. We ran gap2Seq on the reference-based assembly gaps with $k = 19$. For the de novo assembly gaps, we could only get results for a higher $k = 23$, while the implementation could not finish for lower values of k.

Table S7. Comparison of gap filling results for AGapEs and gap2Seq on the London dataset. For each assembly, we divide all gaps into the three respective categories. We count gaps that are filled by both methods and gaps that are only filled by one of both methods. The total value sums up the number of gaps filled by each method.

	reference-based assembly				de novo assembly			
	all	AGapEs	gap2Seq	both	all	AGapEs	gap2Seq	both
consistent	1991	263	3	924	3483	1919	0	886
conflicting	10	3	0	0	7	3	0	0
IS	207	70	0	62	201	76	0	37
total	2208	1322	989		3691	2921	923	
		59,87%	44,79%			79,14%	25,01%	

A.9 Conflicting components

The conflicting components shown in S14 and S17 indicate potential points of genome rearrangements in the phylogeny. Including all these adjacencies prevents from building a linear or circular gene order. We used the aDNA mapping information to select ancestral adjacencies for linearization. Ideally, if one gap is covered by reads, we spot breakpoints in the read mappings to the other gaps in conflict. For the London data set, the first two components in both assemblies are matching, i. e. they coincide in the coordinates of their corresponding extant gaps. These components contain only one adjacency that is supported by the reads each, so we remove all other adjacencies from the set of ancestral adjacencies. For the additional third conflicting component in the reference-based assembly, no adjacency can be supported by the reads and hence all of them are removed in order to reconstruct a set of reliable CARs In the Marseille data set, the conflicting components correspond to the conflicts observed in the de novo assembly for the black death data set. However, in the first connected component, a different adjacency is supported by the reads than in the black death data. This indicates a potential rearrangement breakpoint, however further fragmentation in the set of CARs is preventing an explicit demonstration for that. In the second component, all adjacencies are supported by the reads. Hence, we remove all unsupported adjacencies in the first and all adjacencies in the second component. See Figure S15 for the read coverage of discarded adjacencies.

For the reference-based assembly, the set of ancestral adjacencies can then be ordered into seven Contiguous Ancestral Regions (CARs), while we obtain five CARs for the de novo assembly. We convert the reconstructed sequences of markers back to genome sequences by filling the gaps with the read sequences if possible and resorting to the template sequence otherwise.

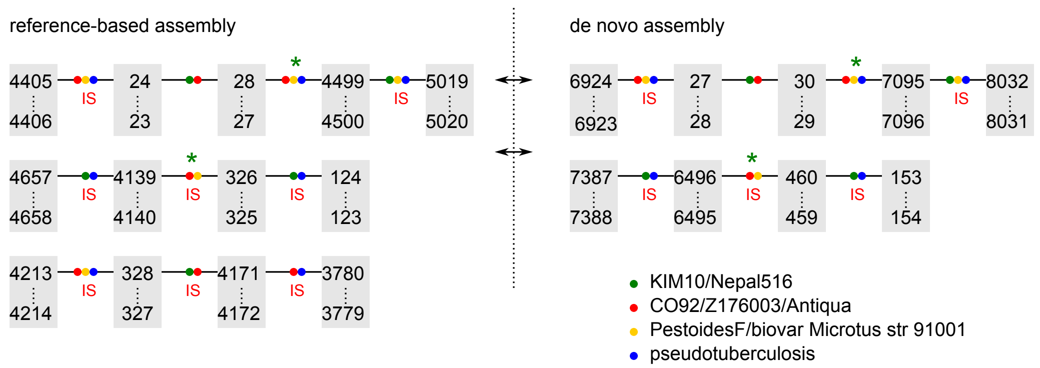

Figure S14. Conflicting components in the set of potential adjacencies for the reference-based assembly and the de novo assembly on the black death data set. Marker with both their extremities are indicated by the grey boxes, while adjacencies are depicted by connecting lines between two extremities. Gaps containing IS sequences are labeled accordingly. The color labels indicate the extant occurrences for each adjacency and hence its conservation in the tree. All gaps that are fully covered by reads and do not contain breakpoints in the mappings are marked by the green stars.

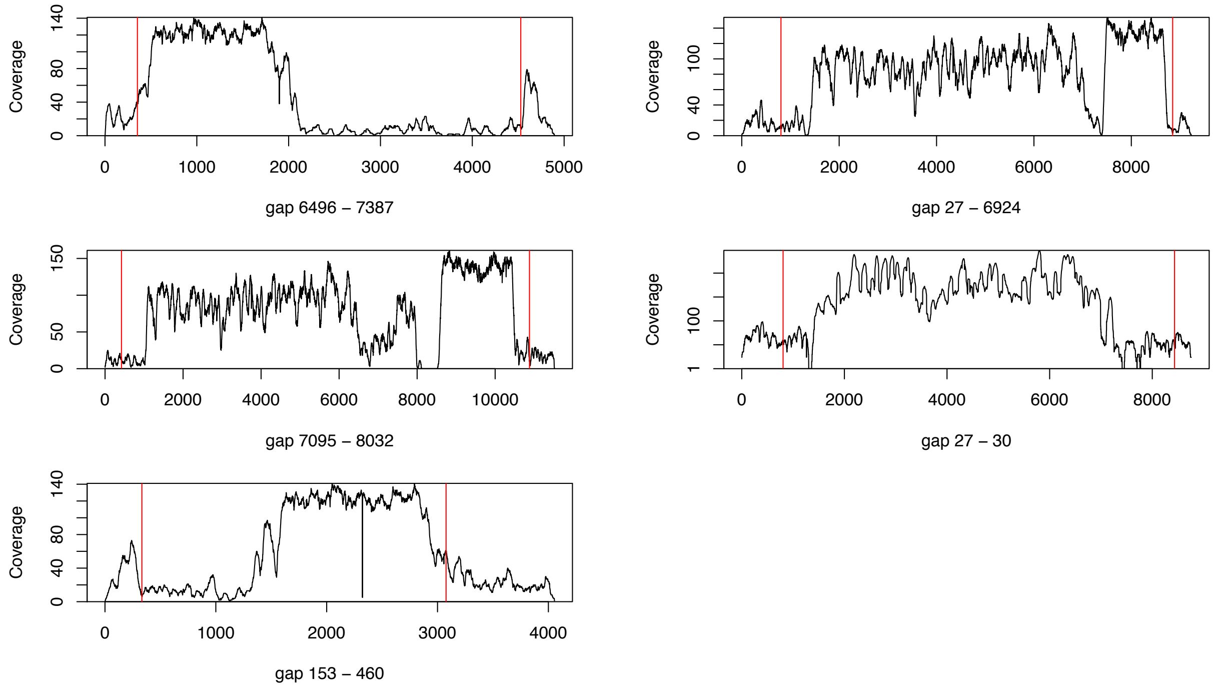

Figure S15. Read coverage for discarded adjacencies in conflicting components for the de novo reconstruction for the London data set. The sequence is flanked by the marker, the gap borders are indicated in red.

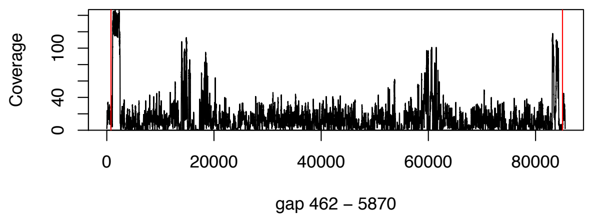

Figure S16. Larger gap between marker for the reconstruction of the London strain that has been removed from the assembly due to insufficient read coverage.

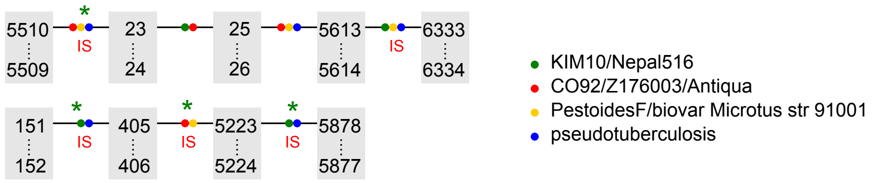

Figure S17. Conflicting components in the set of potential adjacencies for the marseille dataset. Marker with both their extremities are indicated by the grey boxes, while adjacencies are depicted by connecting lines between two extremities. Gaps containing IS sequences are labeled accordingly. The color labels indicate the extant occurrences for each adjacency and hence its conservation in the tree. All gaps that are fully covered by reads and do not contain breakpoints in the mappings are marked by the green stars.

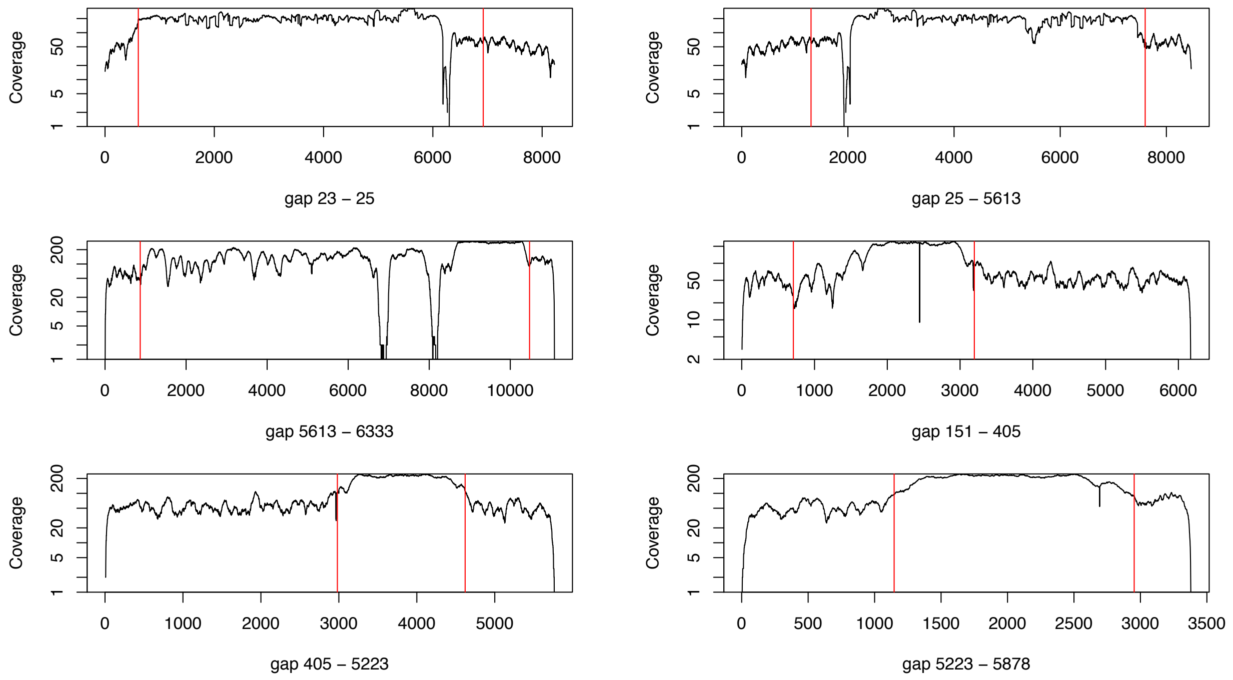

Figure S18. Read coverage for discarded adjacencies in conflicting components for the de novo reconstruction for the Marseille data set. The sequence is flanked by the marker, the gap borders are indicated in red.

A.10 Comparison of improved assemblies for London dataset

We compared the two sets of CARs obtained from both initial assemblies by aligning the resulting genome sequences using MUMmer[7]. As seen in Figure S19, we observe no rearrangements between both resulting sets of CARs, showing that, in terms of large-scale genome organization, the final result does not depend on the initial contig assembly.

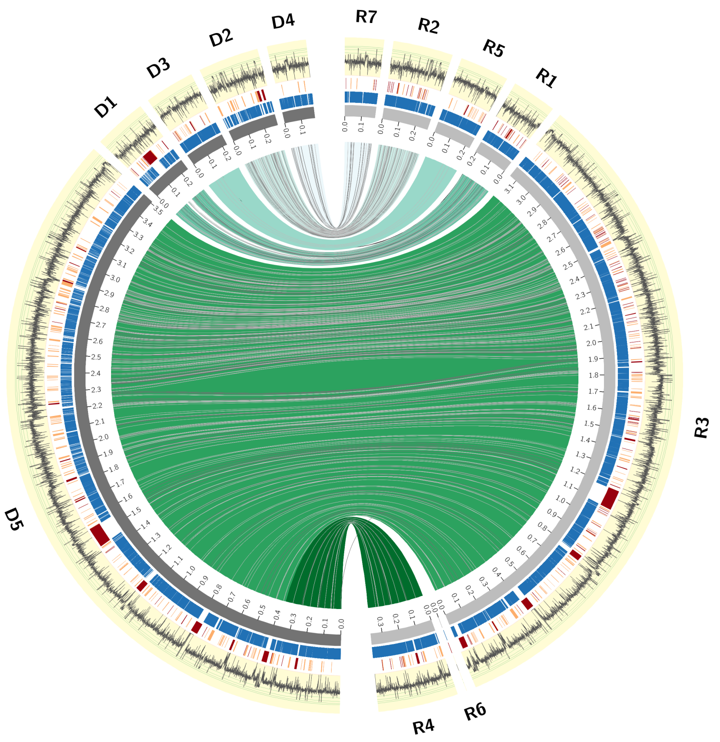

Figure S19. Comparison between the de novo assembly (left) and the reference-based assembly (right) for the London data set. The inner links connect corresponding CARs in the reconstructions. The grey lines indicate substitutions and InDels observed. The positions in both assemblies covered by markers are indicated in blue. All gaps that have IS annotation in the extant genomes are shown in orange. In addition, gaps that are only partially filled or have very unconserved extant gap lengths are indicated in red. Finally, the most outer ring shows the average read coverage in windows of length 200bp in log-scale. Figure done with Circos[6].

In total, only 85,578bp in the reference-based assembly and 88,529bp in the de novo assembly are not covered by any read; however most uncovered regions are rather short (see

We achieve a high similarity between both sets of CARs. While the improved de novo assembly contains a larger amount of filled gap sequences, we align nearly all of both sequences and observe only a low number of SNPs and insertions and deletions between both assemblies (see Figure S19). The observed differences are often located in gaps with low read coverage regions. If short regions in the gaps are only covered by a single read, in order to find a shortest path in the mappings, this read has to be included at all costs and can cause corrections to the template that are not supported by any other read. Further re-sequencing of these regions could clear which variant is present in the ancient genome.

In the improved reference-based assembly, 78.74% of the resulting sequence is defined by markers and hence directly adopted from the initial assembly, while for the improved de novo assembly only 49.88% of the improved assembly is based on marker sequences and a larger part is based on the filled gap sequences. Together with the gaps that have been filled by read sequences, we can say that for the reference-based assembly in total 94.46% and for the de

Table S8. Comparison of improved assemblies on nucleotide level. Both sets of CARs have been corrected with PILON[12], but only corrections of small InDels are kept.

	reference-based	de novo assembly
Aligned sequences	6 (85.71%)	5 (100%)
Total bases	4398441	4441094
Unaligned bases	13145(0.30%)	38702(0.87%)
InDels	216	
Substitutions	389	

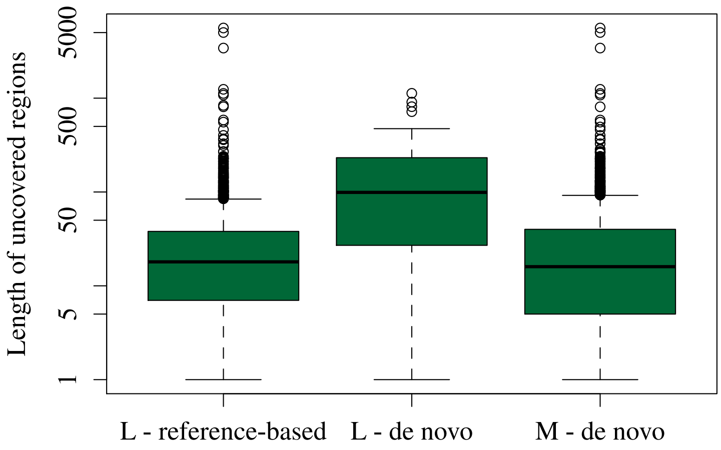

Figure S20. Length distribution of uncovered parts after mapping all reads from both datasets back to the improved reconstructed sequence.

novo assembly in total 95.25% are reconstructed using only the available aDNA reads.

We used BWA[8] to align all reads again to the assembly to assess the amount of uncovered regions in the reconstructed sequences. We keep all optimal mappings for each read. In total, 85578bp in the reference-based assembly and 88529bp in the de novo assembly are not covered by any read. In addition, Figure S20 shows that most uncovered regions are short.

A.11 DCJ distance

The DCJ distance of two genomes A and B represented as sequences of markers is the number of double cut or join operations on adjacencies in A and B. It can be computed based on the adjacency graph $AG(A,B)$, whose vertices display adjacencies and telomeres (marker extremities not contained in any adjacency) of A and B, and there is an edge between vertices that share the same marker extremity. Then the DCJ distance is

$$d_{DCJ}(A,B) = N - (C + I/2) \quad (1)$$

with N being the number of markers, C being the number of cycles and I being the number of odd paths in $AG(A,B)$[1].

A.12 Assembly validation

Table S9. Software parameters for assembly tools

Software	parameters
SPAdes	Multi-cell mode, single reads, read error correction, k automatically selected based on read length
Minia	-abundance-min 3, -kmer-size 21 for Marseille, -kmer-size 19 for London dataset
Ragout	-s sibelia for synteny block decomposition
Medusa	-d to estimate contig distances
Gap2Seq	-k 21

The following table is an extension of Table 1 and describes the assembly statistics for all combinations of SPAdes, minia, ragout and MeDuSa as well as the reference-based assembly with AGapEs described in the appendix.

Table S10. Extended Assembly statistics for both data sets, based on contigs with a minimal length of 500 *bp*. The LAP and CGAL likelihoods have been computed based on all reads mapping to any of the reference sequences.

	Assembly	# contigs	total length	# N's	N50	LAP[5]	CGAL[9]
London	SPAdes	2,555	3,792,691 bp	0	1,888	-11.01048	-6.90196e+08
	Minia	4,183	2,631,422 bp	0	930	-15.69016	-7.98656e+08
	SPAdes-Ragout	1	4,068,385 bp	776,139	-	-12.52232	-4.8192e+08
	Minia-Ragout	2	4,504,786 bp	2020160	4,487,995	-15.72228	-7.86108e+08
	SPAdes-MeDuSa	77	4,333,801 bp	1,917	700,415	-7.97066	-5.00106e+08
	Minia-MeDuSa	9	2,626,626 bp	2074	2,574,520	-15.67916	-7.7175e+08
	ref-AGapEs	6	4,398,314 bp	0	3,147,154	-7.29586	-3.67341e+08
	Minia-AGapEs	5	4,441,104 bp	0	3,511,710	-7.26576	-3.55155e+08
Marseille	SPAdes	3,201	6,072,375 bp	0	4,592	-11.03336	-6.0411e+08
	Minia	3089	3,636,663 bp	0	1,368	-15.05058	-8.71446e+08
	SPAdes-Ragout	2	4,564,323 bp	542,013	4,530,296	-13.34526	-5.84186e+08
	Minia-Ragout	1	3,886,827 bp	1,965,259	-	-16.41699	-9.69013e+08
	SPAdes-MeDuSa	2155	6,052,372 bp	618	1,643,585	-10.88342	-6.12532e+08
	Minia-MeDuSa	125	3,638,125 bp	1462	2,695,392	-15.03495	-8.42249e+08
	Minia-AGapEs	6	4,350,872 bp	0	3,459,919	-8.05526	-4.32647e+08

Table S11. Gene predictions for London strain and *Yersinia pestis CO92* computed with prokka[11].

	Minia +AGapEs	SPAdes +Ragout +Gap2Seq	Bos	Minia	CO92
CDS	3943	4027	3376	2023	4090
rRNA	16	0	1	0	19
tRNA	71	18	30	0	69
tmRNA	1	1	0	0	1
repeat_region	3	1	0	0	3

Table S12. Gene predictions for Marseille strain and *Yersinia pestis CO92* computed with prokka[11].

	Minia +AGapEs	SPAdes +Ragout +Gap2Seq	Minia	CO92
CDS	3876	3924	2997	4090
rRNA	15	0	0	19
tRNA	68	51	4	69
tmRNA	1	1	0	1
repeat_region	3	1	0	3

References

1. Anne Bergeron, Julia Mixtacki, and Jens Stoye. A unifying view of genome rearrangements. In *International Workshop on Algorithms in Bioinformatics*, pages 163–173. Springer, 2006.

2. Kirsten I Bos, Alexander Herbig, Jason Sahl, Nicholas Waglechner, Mathieu Fourment, Stephen A Forrest, et al. Eighteenth century yersinia pestis genomes reveal the long-term persistence of an historical plague focus. *eLife*, page e12994, 2016.

3. Kirsten I. Bos, Verena J. Schuenemann, G. Brian Golding, Hernán A. Burbano, Nicholas Waglechner, Brian K. Coombes, et al. A draft genome of Yersinia pestis from victims of the Black Death. *Nature*, 478:506–510, 2011.

4. Rayan Chikhi and Guillaume Rizk. Space-efficient and exact de Bruijn graph representation based on a Bloom filter. *Algorithms for Molecular Biology*, 8:1, 2013.

5. Mohammadreza Ghodsi, Christopher M Hill, Irina Astrovskaya, Henry Lin, Dan D Sommer, Sergey Koren, and Mihai Pop. De novo likelihood-based measures for comparing genome assemblies. *BMC research notes*, 6(1):334, 2013.

6. Martin I Krzywinski, Jacqueline E Schein, Inanc Birol, Joseph Connors, Randy Gascoyne, Doug Horsman, Steven J Jones, and Marco A Marra. Circos: An information aesthetic for comparative genomics. *Genome Research*, 2009.

7. Stefan Kurtz, Adam Phillippy, Arthur L Delcher, Michael Smoot, Martin Shumway, Corina Antonescu, and Steven L Salzberg. Versatile and open software for comparing large genomes. *Genome Biology*, 5:R12, 2004.

8. Heng Li and Richard Durbin. Fast and accurate short read alignment with Burrows–Wheeler transform. *Bioinformatics*, 25:1754–1760, 2009.

9. Atif Rahman and Lior Pachter. CGAL: computing genome assembly likelihoods. *Genome biology*, 14(1):R8, 2013.

10. Ashok Rajaraman, Eric Tannier, and Cedric Chauve. FPSAC: fast phylogenetic scaffolding of ancient contigs. *Bioinformatics*, 29:2987–2994, 2013.

11. Torsten Seemann. Prokka: rapid prokaryotic genome annotation. *Bioinformatics*, page btu153, 2014.

12. Bruce J Walker, Thomas Abeel, Terrance Shea, Margaret Priest, Amr Abouelliel, Sharadha Sakthikumar, et al. Pilon: an integrated tool for comprehensive microbial variant detection and genome assembly improvement. *PLoS One*, 9(11):e112963, 2014.

13. Derrick E Wood and Steven L Salzberg. Kraken: ultrafast metagenomic sequence classification using exact alignments. *Genome biology*, 15(3):R46, 2014.

14. Daniel R Zerbino and Ewan Birney. Velvet: algorithms for de novo short read assembly using de bruijn graphs. *Genome research*, 18(5):821–829, 2008.
